# Reduced Alternating Gaze During Social Interaction in Infancy is Associated with Elevated Symptoms of Autism in Toddlerhood

**DOI:** 10.1007/s10802-017-0388-0

**Published:** 2018-03-12

**Authors:** Emilia Thorup, Pär Nyström, Gustaf Gredebäck, Sven Bölte, Terje Falck-Ytter, the EASE Team

**Affiliations:** 1 Uppsala Child and Baby Lab, Department of Psychology, Uppsala University, Uppsala, Sweden; 2 Karolinska Institutet Center of Neurodevelopmental Disorders (KIND), Pediatric Neuropsychiatry Unit, Department of Women’s & Children’s Health, Karolinska Institutet, Stockholm, Sweden; 3 BUP Stockholm, Center for Psychiatry Research, Stockholm County Council, Stockholm, Sweden

**Keywords:** Autism, Joint attention, Communication, Eye tracking, Motivation, Infant siblings

## Abstract

In typical development, infants often alternate their gaze between their interaction partners and interesting stimuli, increasing the probability of joint attention toward surrounding objects and creating opportunities for communication and learning. Children with Autism Spectrum Disorder (ASD) have been found to engage less in behaviors that can initiate joint attention compared to typically developing children, but the role of such atypicalities in the development of ASD during infancy is not fully understood. Here, using eye tracking technology in a live setting, we show that 10-month-olds at high familial risk for ASD engage less in alternating gaze during interaction with an adult compared to low risk infants. These differences could not be explained by low general social preference or slow visual disengagement, as the groups performed similarly in these respects. We also found that less alternating gaze at 10 months was associated with more social ASD symptoms and less showing and pointing at 18 months. These relations were similar in both the high risk and the low risk groups, and remained when controlling for general social preference and disengagement latencies. This study shows that atypicalities in alternating gaze in infants at high risk for ASD emerge already during the first 10 months of life - a finding with theoretical as well as potential practical implications.

## ᅟ

Joint attention (JA) – which can be defined as the sharing of attention between individuals and an object (Bruner 1975; Scaife and Bruner 1975) - plays an important role for learning and social interaction early in life. Young infants respond to others’ joint attention bids by following their gaze and pointing gestures (Schmitow et al. 2016; Corkum and Moore 1998). This allows them to experience what others see, and facilitates learning about objects and events in the world. After a while, infants start directing others’ attention in order to share their own experiences, first by alternating their gaze between an interaction partner and an object, and later by the use of gestures such as pointing (Beuker et al. 2013; Carpenter et al. 1998). In other words, the infants no longer merely respond to joint attention; they initiate it as well. Although initiating joint attention (IJA) and responding to joint attention (RJA) may seem like two sides of the same coin, evidence suggests that they represent partially different processes. For example, longitudinal correlations within each type of JA are higher than correlations between the two types of JA at a given time point (Mundy et al. 2007). IJA and RJA also follow different developmental trajectories. Whereas RJA begins to emerge between 3 and 4 months of age (Gredebäck et al. 2010; D'Entremont et al. 1997; Perra and Gattis 2010), IJA behaviors typically emerge between 8 and 13 months (Beuker et al. 2013). Also at the neural level, there is some evidence that the two types of JA are supported by partially different systems. It has been suggested that RJA relies primarily on a posterior attention system that regulates involuntary attention, whereas IJA seems to be supported by a later developing anterior network, controlling volitional attention (Mundy et al. 2000, 2009; Van Hecke et al. 2007). Further, IJA but not RJA is associated with activity in reward-related brain areas (Schilbach et al. 2010; Gordon et al. 2013) suggesting a link between IJA and motivation.

It is well known that children with autism spectrum disorder (ASD) engage less in JA. Parental report of JA behaviors at 8 months has been shown to predict diagnostic status at 7 years (Veness et al. 2014), thus suggesting that JA may be one of the earliest domains where infants with later ASD differ from other infants. Deficits in IJA are considered more impairing and tend to persist longer in the development of autistic children than deficits in RJA (Mundy et al. 1994; Sigman and Ruskin 1999; Gotham et al. 2007; Lord et al. 2000). The social motivation account (Chevallier et al. 2012) proposes early diminished social motivation as a causal factor behind the later occurring social-cognitive impairment observed in autistic individuals. If infants who will later be diagnosed with ASD find sharing their experiences with others less rewarding, they can be expected to initiate JA to a lesser extent than other infants. Considering the important role JA plays in social learning and interaction, less initiation of JA would likely have an attenuating effect on several aspects of development. It is therefore imperative that we learn more about the early development of IJA in ASD.

### Measuring Different Types of IJA Behaviors and Their Relation to Each Other and to ASD

Young children may attempt to direct others’ attention by the use of a variety of behaviors, and studies of IJA typically measure gestural behaviors such as pointing and showing as well as eye contact behaviors. Some (e.g. Stone et al. 2000; Wetherby and Prizant 2002) include vocal behaviors as well. One of the earliest means by which infants initiate JA is by looking back and forth between an interaction partner and an object or event. Alternating gaze with the purpose of attention sharing typically emerges around 8–9 months of age (Beuker et al. 2013), while gestural IJA behaviors such as pointing and showing typically first emerge between 10 and 13 months (Beuker et al. 2013; Carpenter et al. 1998). Focusing on alternating gaze therefore entails a possibility to detect differences earlier than what is possible when gestural IJA behaviors are measured. However, some studies have indicated that it is predominantly in terms of the gestural behaviors that the performance of autistic children differs from that of typically developing children (Chiang et al. 2008; Mundy et al. 1994). In a study of children with ASD, Pickard and Ingersoll (2015) assessed eye contact based and gestural IJA separately. It was discovered that the two types of IJA were unrelated to each other, and that only gestural IJA was associated with language, RJA and imitation. This led the authors to suggest that eye contact based IJA (such as alternating gaze) may be a less optimal measure in ASD research. It is important to note though, that the children in the study by Pickard and Ingersoll (2015) were 2–7 years old, and therefore had reached an age when gestural behaviors such as pointing and showing have long been part of most children’s repertoires. It is thus possible that these behaviors as well as language had in part replaced alternating gaze as a means of initiating JA.

### Prospective Studies of IJA

ASD is rarely diagnosed before the age of 24–36 months, which implies that assessing IJA in diagnosed children does not capture possible differences in terms of early development. Because 7–20% of younger siblings of children with ASD are expected to receive an ASD diagnosis themselves (Messinger et al. 2015; Ozonoff et al. 2011; Gronborg et al. 2013), studying younger siblings longitudinally has proven an important means of gaining insight in the early development of ASD.

Several studies have indicated that receiving an ASD diagnosis is associated with lower levels or lower growth rates of IJA during the second year of life (Macari et al. 2012; Landa and Garrett-Mayer 2007; Rozga et al. 2011; Yoder et al. 2009). Also, high risk (HR) infants, i.e. those with an older sibling with ASD, have been found to engage in fewer IJA behaviors than low risk (LR) infants during this time period (Goldberg et al. 2005; Cassel et al. 2007, but see Yirmiya et al. 2006). Only two of these studies (Macari et al. 2012; Landa and Garrett-Mayer 2007) measured alternating gaze specifically. The remaining studies measured IJA as a broader category, including other communicative behaviors as well. It is therefore not possible to conclude whether the results were driven by differences in for example gestural behaviors, or whether alternating gaze specifically differs between groups.

To our best knowledge, only one study has prospectively investigated IJA before the first birthday (Ibanez et al. 2013). This study showed that the level of IJA at 8 months predicted diagnostic outcome (ASD or no diagnosis) at 30 months in a HR sample. As with the majority of the studies mentioned above however, this study did not focus specifically on alternating gaze, but measured IJA in a broader sense. There is however one retrospective study (Clifford and Dissanayake 2009) that has reported a relation between alternating gaze as assessed using home videos recorded during the children’s first 11 months, and a measure of social responsiveness at 4 years.

One issue that prospective studies can help clarifying is the timing of the onset of difficulties in alternating gaze in infants who later develop autism. This question is theoretically important because, as pointed out by Jones et al. (2014), current research cannot tell us whether atypical alternating gaze could be considered an early risk factor itself, or a later consequence of other risk factors. Considering that most autistic symptoms emerge during the second year of life (Jones et al. 2014), more studies of alternating gaze before the first birthday are needed.

### Variables that may Influence Alternating Gaze

The majority of the studies assessing IJA have used instruments where interaction between the child and an experimenter is coded by a human observer based on a predefined protocol ((e.g. the Early Social Communication Scales (ESCS; Mundy et al. 1996) or the Screening Tool for Autism in Two-year-olds (STAT; Stone et al. 2000)). Although this is well suited for measuring gestural IJA such as pointing, analyses of gaze alternations are likely to benefit from the use of more precise methods. Eye tracking has the advantage of rendering exact measures of gaze behavior that allow for various analyses of visual attention to be conducted in order to test different hypotheses (Falck-Ytter et al. 2013). It can therefore be used to generate measures of factors that may potentially have an impact on IJA behaviors. As previously mentioned, less IJA engagement could be a consequence of a decreased preference for social stimuli in general, which could be assessed via looking times at social and non-social stimuli. It has also been shown that infants with later ASD may have difficulties disengaging their attention from one stimulus to another (Elsabbagh et al. 2013; Elison et al. 2013; Zwaigenbaum et al. 2005), which could result in less alternating gaze. The possibility therefore remains that the lower frequency of alternating gaze that has been found in infants with later ASD in some studies (Ibanez et al. 2013; Landa and Garrett-Mayer 2007; Macari et al. 2012) is in fact a direct consequence of disengagement difficulties. This could also potentially explain the lack of a concurrent correlation between different types of IJA detected in autistic preschoolers (Pickard and Ingersoll 2015), since disengagement difficulties would be expected to influence gaze behavior more than gestural behaviors. To our knowledge, only one study has investigated the relation between IJA and the aforementioned factors. In a cross-sectional study of three-year-olds with ASD, Schietecatte et al. (2012) reported a negative relation between disengagement latencies and eye contact based IJA (including alternating gaze), whereas a non-significant trend was found between the former and social preference.

Despite the previously mentioned advantages of eye tracking, the most frequently used application of the method has a clear disadvantage in that it uses pre-recorded stimuli (images or videos). Humans look at others differently when watching them on video as compared to when watching them in real life (Foulsham et al. 2011; Laidlaw et al. 2011), and it has been shown that measuring live interaction evokes different responses than displaying pre-recorded stimuli, both in terms of brain activation (Redcay et al. 2010; Schilbach et al. 2010; Shimada and Hiraki 2006) and behavior (Risko et al. 2012; Freeth et al. 2013). Video stimuli can therefore not be considered optimal when the focus is on social behaviors such as IJA. Instead, eye tracking with live stimuli should be considered.

### Aims of the Study

In the current study, eye tracking technology was used in a live setting, allowing infants’ gaze to be recorded while they were interacting with another (adult) person in an IJA eliciting task. When they reached 18 months, an age where autistic symptoms are detectable in several domains (Jones et al. 2014), the Autism Diagnostic Observation Schedule-Toddler Module (ADOS-T; Lord et al. 2012) was administered in order to obtain measures of ASD symptom level, as well as gestural IJA.

First, we tested the hypothesis that the HR group would display less alternating gaze behavior than the LR group at 10 months of age. Second, the relation between alternating gaze at ten months and later ASD symptoms was assessed. Here, the hypothesis was that the extent to which infants engaged in alternating gaze at 10 months would be negatively associated with ASD symptomatology at 18 months. Next, in order to explore the potential mechanisms behind alternating gaze and how they may differ between groups, measures of general social preference as well as disengagement ability were included. No a priori hypotheses were formed regarding these measures. Finally, we wanted to assess the validity of alternating gaze as a measure of IJA, by investigating its relation to later occurring gestural IJA behaviors (showing and pointing). It was predicted that alternating gaze at 10 months would be positively associated with showing and pointing at 18 months.

## Methods

### Participants

A total of 67 infants participated in the study (final sample after exclusion; for participant characteristics, see Table 1). Fifty-one infants (29 girls, 22 boys) were HR infants, having at least one older full sibling with ASD. Sixteen infants (6 girls, 10 boys) were LR infants, having at least one older typically developing full sibling and no family history of ASD. Data from an additional 9 infants (6 HR and 3 LR) was collected but excluded due to poor data quality or the infants not contributing enough data (see Analysis section). All infants were part of an ongoing longitudinal project (Early Autism Sweden; EASE; www.smasyskon.se) following HR and LR infants from 5 to 72 months. The HR group was recruited through advertisements, the project’s webpage and clinical units. The LR group was recruited from a database of families who had indicated interest in participating in research with their infants. Both groups consisted primarily of infants from the larger Stockholm, Sweden, area. Exclusion criteria were pre-term birth (< 36 weeks) and confirmed or suspected medical problems, including visual/auditory impairment. There was no difference between HR and LR groups in terms of socioeconomic status based on family income and education level. The developmental level of the infants was assessed using the Mullen Scales of Early Learning (MSEL; Mullen 1995) at each visit, and did not differ between groups. The diagnosis of the older sibling was confirmed through inspection of obtained child psychiatric or pediatric records (more than 70% of all assessments included the ADOS (Lord et al. 2000) and/or the Autism Diagnostic Interview-Revised (ADI-R; Rutter et al. 2003)). The study was approved by the Regional Ethical Review Board in Stockholm and conducted in accordance with the standards specified in the 1964 Declaration of Helsinki. All parents provided written informed consent.

**Table 1:** Participant characteristics by group, final samples (M/SD)

Measure	HR group *N* = 51 (29 girls)	LR group *N* = 16 (6 girls)	Pairwise comparison *p*-value
Age at 10 month assessment	10.45/0.43	10.37/0.55	> 0.25^a^
MSEL Early Learning Composite 10 months	99.92/13.50	101.44/11.64	> 0.25^a^
Age at 18 month assessment	18.51/0.76	18.51/0.94	> 0.25^a^
MSEL Early Learning Composite 18 months	97.29/15.67	97.75/13.54	> 0.25^a^
SES^b^	- 0.02/0.84	0.08/084	> 0.25^a^
ADOS-T Total CSS 18 months	3.18/1.74	2.06/1.53	0.009^c^
ADOS-T SA CSS	3.24/1.91	1.94/1.73	0.004^c^
ADOS-T RRB CSS	3.98/2.09	3.31/2.12	0.204^c^
ADOS-T Showing 18 months	0.92/0.91	0.44/0.81	0.03^c^
ADOS-T Pointing 18 months	0.71/0.81	0.38/0.50	0.157^c^

^a^Independent samples t-test

^b^Socioeconomic status calculated on the basis of parental education and income (equal weighting), expressed as a z-score (for this measure, *N* = 50 in the HR group and 15 in the LR group since two families did not disclose this information)

^c^Mann-Whitney U Test

### Stimuli and Procedure

#### 10-Month Visit

The participants took part in a comprehensive assessment, typically spending 4–5 h in the lab. This study includes data from the eye tracking experiment and the MSEL (Mullen 1995).

**Fig. 1:**
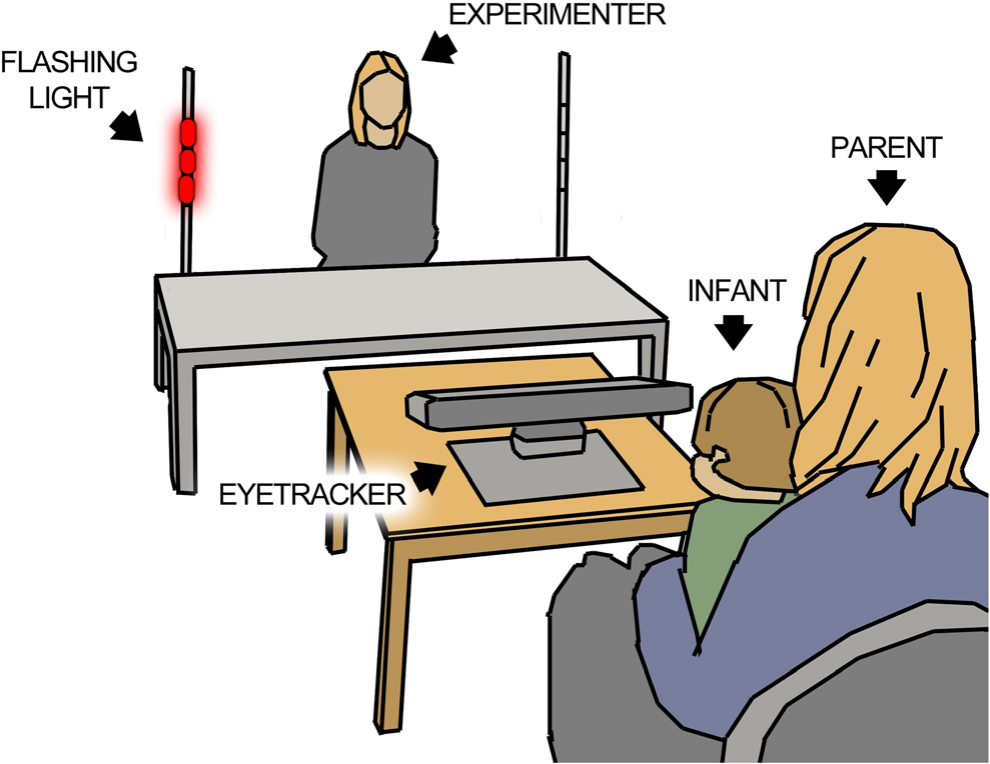
Sketch of the experimental setting. The infant was seated at a distance of 200 cm from the experimenter. The infant’s gaze was recorded by a Tobii TX300 eye tracker (placed at the table between the infant and experimenter). The infant’s behavior as well as the scene area was recorded by two video cameras (not visible in the sketch)

The eye tracking was administered early during the visit. The infant was seated on the lap of the parent, at a distance of 200 cm from the experimenter, who was seated at a low table (see Fig. 1). At each side of the table was an oblong transparent lamp. The experimenter controlled the lamps with two remote controls, hidden beneath the table and thus invisible to the child. A Tobii TX300 eye tracker, placed on a table in front of the infant, was used to record the infant’s gaze with a sample rate of 120 Hz. Two video cameras recorded the behavior of the infant and the stimulus area. Before the session, a five point calibration procedure was conducted. The experimenter moved a squeaky toy across predefined calibration points, making the toy emit a sound at each point to attract the attention of the infant. The procedure was repeated if necessary until calibration was satisfactory (determined via inspection of online gaze replay on a monitor in the background of the room). The live eye tracking session comprised a variety of different tasks and lasted approximately 10–15 min in total. Data from the IJA task and the disengagement task (described below) will be reported in the present paper, as well as a measure of general social preference that was obtained during all other parts of the session. The IJA task always took place at the end of the session, and the disengagement task was conducted in the middle of the session.

The IJA task consisted of four trials and lasted approximately 1–1.5 min in total. The experimenter started each trial by attracting the infant’s attention. Once that was achieved the experimenter turned on the lamp on his/her right side and lights started to flash, changing color approximately every second. The switch controlling the lamp was out of sight for the infant, who was not aware that the lights were controlled by the experimenter. While the lights were flashing, the experimenter sat still looking at the infant, intermittently speaking in a quiet tone of voice. This was to provide the infant with an opportunity to initiate joint attention toward the lamp. If the child made an explicit attempt to direct the experimenter’s attention to the lights, e.g. by pointing or vocalizing (e.g. saying “there” while looking toward the lights), the experimenter responded by turning toward the lights and commenting on them. The rationale behind this was to not upset the infant or possibly extinguish IJA behaviors by being unresponsive. The lights were flashing for a total of 10 s. The lamp was then turned off, and the second trial was initiated. All four trials were identical, except that the lights on the experimenter’s right side were activated for the first and third trials, and the lights on the left side were activated for the second and fourth trials.

In order to check for differences in the ability to disengage from one stimulus to another, a modified version of the disengagement task from the Autism Observation Scale for Infants (AOSI; Bryson et al. 2008) was included in the eye tracking session. The experimenter shook a rattle approximately 20 cm to the right from his/her face, keeping another rattle hidden underneath the table. Once the infant looked at the first rattle, the second was presented and shaken at the left side of the experimenter’s face. In total six disengagement trials were conducted, alternating between the left and right sides.

The experiment was performed by six individual experimenters (2 males, 4 females). In order to minimize the influence of individual experimenters, each experimenter was trained according to a written protocol and a video template. The MSEL (Mullen 1995) was administered after the eye tracking experiment.

#### 18-Month Visit

Similar to the 10-month visit, the children spent a total of 4–5 h in the lab taking part in a variety of tasks and assessments. This study includes data from the MSEL and the ADOS-T. The MSEL was administered early during the day, and the ADOS-T was typically administered midday after a lunch break. The ADOS (Lord et al. 1999) is a widely used diagnostic instrument in autism assessment, and recommended as first choice instrument by several international clinical guidelines (e.g. NICE.org.uk). Considering the young age of the participants, the Toddler Module (Lord et al. 2012) was used, which has been developed to assess behaviors indicative of autism in children younger than 30 months, with low verbal skills and a developmental age of at least 12 months. The ADOS-T was administered by three experienced clinical psychologists. One of the clinicians had achieved formal research reliability, and was supervising the work of the other two, who both had extensive clinical experience in using the instrument.

### Analysis

Data text files were exported from the Tobii Studio Software. Analyses were conducted in MATLAB r2015b (MathWorks Inc., Natick, MA), using the TimeStudio project framework, a MATLAB-based open access analysis tool for time series data (version 3.15; timestudioproject.com; Nyström et al. 2016) and SPSS (SPSS Inc., Chicago, IL).

#### IJA Task at 10 Months

For each individual recording, four areas of interest (AOIs) were defined from which gaze data was extracted. One covered the face of the experimenter, two covered the lamps and the fourth AOI covered the entire scene area. The face AOI subtended 11 by 11 visual degrees and the lamp AOIs each subtended 14 by 17 visual degrees. A fixation filter (Tobii Fixation Filter) with a velocity threshold of 35 pixels/window and a distance threshold of 35 pixels was applied. Because manual inspection revealed that on some trials the lights were activated slightly shorter than 10 s, only data from the first 9 s of light activation time were analyzed for all trials. Only trials with at least 50% gaze data during light activation were included in the analysis. Further, we required at least two valid trials (out of four) from each child. This resulted in an average of 3.20 (SD = 0.87) usable trials in the HR group and 3.31 (SD = 0.87) usable trials in the LR group (*p* > 0.25). As the primary measure of alternating gaze behavior, we used the mean number of gaze alternations between the face of the experimenter and the flashing lights (i.e. the sum of gaze alternations from lights to face and face to lights) per trial. In order to control for general differences in looking patterns and durations, the total number of fixations on the scene area and the total looking times in the different AOIs were also compared across groups.

#### Disengagement Task at 10 Months

Two AOIs covering the two rattles were defined, subtending 6 by 18 visual degrees each. The dependent measure was the time it took the infant’s gaze to enter the AOI of the second rattle once it became visible to the infant. All trials in which the infants made a predictive gaze shift (i.e. disengaged from one rattle before the other was presented) were excluded from the analysis. Each infant had to contribute at least two valid disengagement trials in order to be included, which resulted in 57 infants (45 HR and 12 LR) contributing data to this analysis. The mean number of usable trials was 3.98 (SD = 1.84) in the HR group and 3.80 (SD = 2.21) in the LR group (*p* > 0.25).

#### Social Preference at 10 Months

In order to assess possible differences in how much relative time the infants spent looking at the experimenter, a social preference quotient was calculated during the entire live eye tracking session *except* the IJA task and the disengagement task. The eye tracking session builds on naturalistic social interaction between the experimenter and the infant. It includes a multitude of components such as the experimenter playing with and looking at a variety of toys; the experimenter talking to and calling the infant’s name etc. Two tasks are described elsewhere (Thorup et al. 2016; Nyström et al. 2017). The present objects were at all times kept out of the experimenter’s face area, which allowed us to calculate a ratio of relative face looking. The total amount of the time the infants spent looking at the experimenter’s face was divided by the total amount of time they spent looking anywhere on the scene area, including the experimenter and the various present objects. Higher values would thus indicate a higher preference for the experimenter over the non-social elements. Relative looking times at social vs. non-social stimuli have previously been used to evaluate social interest in infants and toddlers at risk for ASD (e.g. Pierce et al. 2016; Chawarska et al. 2013). To be included in the social preference analysis the infants were required to look at the scene (contribute valid gaze data) for at least 25% of the entire session, which resulted in the inclusion of 65 infants (50 HR, 15 LR).

#### ADOS-T Measures at 18 Months

Given the young age of the children, we did not classify different outcome groups, but rather investigated autism symptoms quantitatively, operationalized as performance on the ADOS-T. As a general measure of ASD symptomatology the Total Social Affect and Restricted Repetitive Behavior Score was used. In order to test whether alternating gaze may be differently related to the social-communicative domain and the restricted and repetitive behavior (RRB) domain, we also used the Social Affect (SA) and RRB scores separately. For these three measures, rather than using the raw scores we used the calibrated severity scores (CSS), as suggested by Esler et al. (2015). CSS have been shown to result in more uniform distributions across age and language level and to be less influenced by non-ASD specific child characteristics compared to raw scores (Esler et al. 2015; Gotham et al. 2009). CSS range from 1 to 10, with higher values indicating more symptoms and a higher degree of concern. In order to investigate the relation between alternating gaze at 10 months and later gestural IJA, the items showing and pointing were used. Both items are scored on a four point scale from 0 to 3 with lower values indicating higher incidence and quality of the behaviors. The ADOS-T also contains an item where alternating gaze is scored. Unfortunately, our data did not contain enough variation for analyses regarding this item to be conducted.

#### Statistical Analysis

**Fig. 2:**
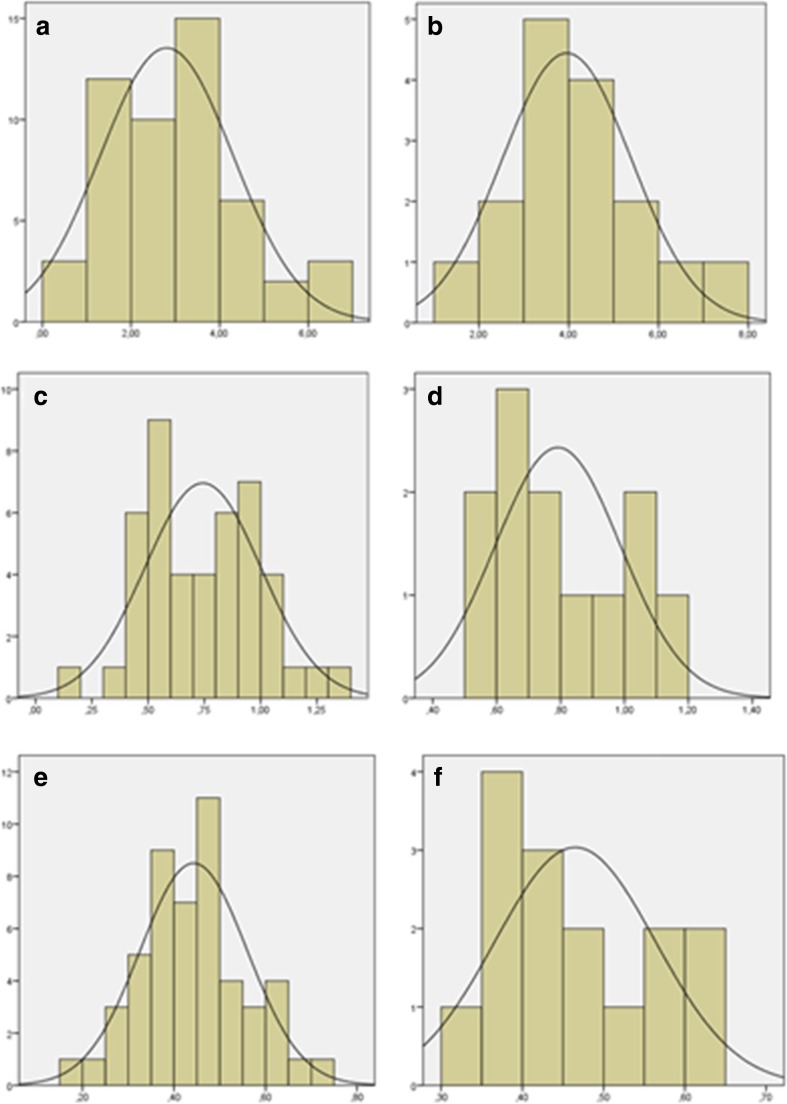
Histograms showing distributions for the experimental variables per group. a) alternating gaze, HR group; b) alternating gaze, LR group; c) disengagement latency, HR group; d) disengagement latency, LR group; e) social preference, HR group; f) social preference, LR group. Note that none of the distributions differs significantly from normality according to the Kolmogorov-Smirnov test

The ADOS-T variables were not normally distributed and non-parametric tests (Spearman correlations and multiple linear regression analysis with bootstrap for coefficients (1000 samples)) were therefore used to assess relations and group comparisons involving these variables unless otherwise specified. All other variables were normally distributed with equal variances across groups, and thus analyzed by the use of parametric statistics. For distributions of the experimental variables, see Fig. 2*.* The gender distribution did not differ significantly between groups, *X*(1) = 2.23, *p* = 0.136, and no differences between boys and girls were found on any of the outcome variables, all *p*-values ≥0.216. Gender was therefore not used as a covariate.

## Results

### High vs Low Risk Comparisons at 10 Months

**Fig. 3:**
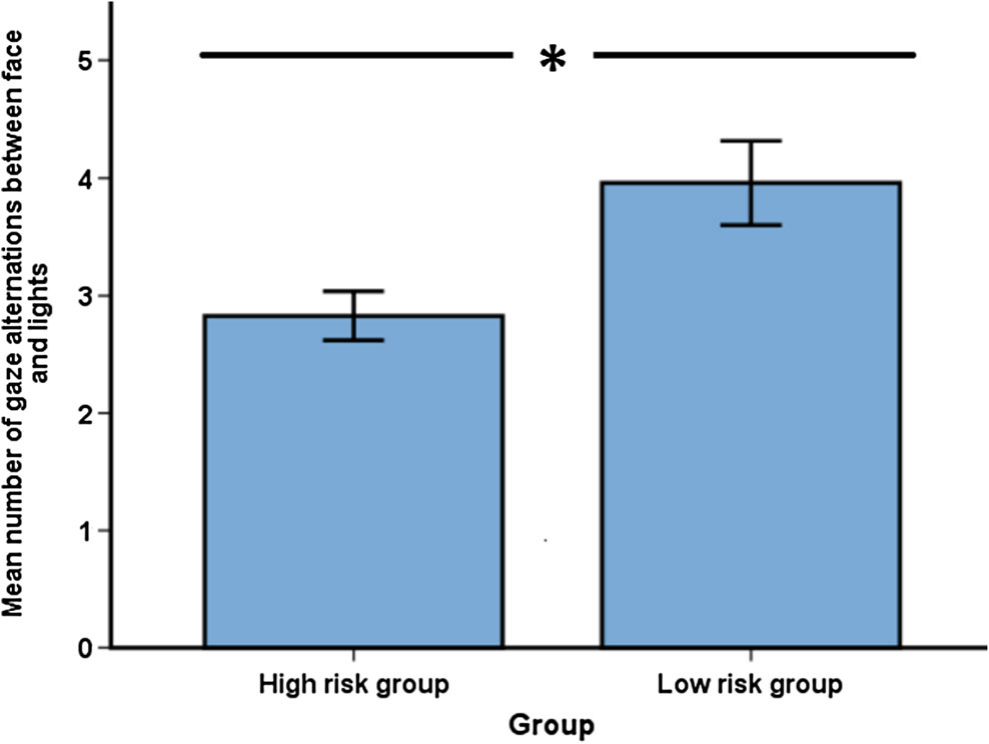
Number of gaze alternations made between the face of the experimenter and the lights per second. Error bars represent standard errors. * *p* < 0.05

An independent samples t-test on the mean number of gaze alternations between experimenter and lights revealed a group difference, *t*(65) = 2.66, *p* = 0.012, *d* = 0.77, with the LR group making more such gaze alternations than the HR group (see Fig. 3). The groups did not differ in terms of the duration of time they spent looking at either the face of the experimenter, *t*(65) = 0.84, *p* > 0.25, *d* = 0.24, or the lights, *t*(65) = 0.17, *p* > 0.25, *d* = 0.05 (for descriptive statistics for these and the following eye tracking measures, see Table 2). The mean number of fixations on the scene area also did not differ between groups, *t*(65) = −0.01, *p* > 0.25, *d* = 0.002. In order to check for possible effects of experimenter, we compared the alternating gaze performance of the children who saw the experimenter who tested the largest amount of infants (48%) to the performance of the remaining infants (who each saw 1 of the 5 other experimenters). No difference was found between the groups, *t*(65) = − 1.20, *p* = 0.233, *d* = 0.29.

**Table 2:** Descriptive statistics by group for 10-month eye tracking measures (M/SD)

Measure	HR group *N* = 51 (29 girls)	LR group *N* = 16 (6 girls)
Looking time (s) at model’s face	2.30/1.45	2.65/1.48
Looking time (s) at lights	4.41/1.55	4.49/1.45
N fixations on scene area	13.32/6.05	13.31/4.91
Disengagement latency^a^ (s)	0.74/0.25	0.79/0.20
Social preference^b, c^	0.44/0.12	0.47/0.10

^a^Based on 45 HR and 12 LR infants

^b^Based on 50 HR and 15 LR infants

^c^Looking time at model divided by total looking time on scene area

The results from the disengagement task showed that the groups did not differ in terms of the time it took the infants to disengage their attention from one active stimulus to another, *t*(55) = 0.62, *p* > 0.25, *d* = 0.22. A significant negative correlation between disengagement and alternating gaze was found in the LR group, *r*(10) = − 0.77, *p* = 0.004, suggesting that longer latencies to disengage were associated with less alternating gaze. In the HR group, a marginally significant trend in the same direction was detected, *r*(43) = − 0.28, *p* = 0.062.

No group difference was found in terms of social preference, *t*(63) = 0.69, *p* = > 0.25, *d* = 0.27 (independent samples t-test). Social preference and alternating gaze did not correlate in either the HR group, *r*(48) = 0.23, *p* = 0.109, or the LR group, *r*(13) = −0.23, *p* > 0.25.

### Longitudinal Relations Between 10-Month Measures and 18-Month Symptom Level

In order to test whether alternating gaze could predict later symptom severity, a hierarchical multiple regression was conducted. Total ADOS-T score was entered as the dependent variable, and as independent variables, alternating gaze and group were added in the first block, and the interaction between group and alternating gaze was added in the second block. Bootstrapping was used to account for the skewed distribution of the ADOS-T scores. The first model was significant, *R*^*2*^ = 0.16, *F*(2,64) = 6.29, *p* = 0.003, and showed that alternating gaze at 10 months, but not group status, made a significant contribution to 18 month ADOS-T scores. The interaction term had no significant individual contribution to the ADOS-T scores and was thus excluded from the model (Table 3). With no other factors included in the model, alternating gaze alone explained 14% of the variance in ADOS-T scores, *p* = 0.002.

**Table 3:** Regression table displaying the results of analyses with alternating gaze and group as independent variables, and Total ADOS-T scores, ADOS-T SA scores, and ADOS-T RRB scores as dependent variables

Dependent variable		B	SE B	β
Total ADOS-T score	Constant	3.46	0.67	
	Alternating gaze	−0.35	0.14	−0.31*
	Group	0.71	0.49	0.18
ADOS-T SA score	Constant	3.55	0.74	
	Alternating gaze	−0.41	0.15	−0.33*
	Group	0.84	0.54	0.19
ADOS-T RRB score	Constant	3.25	0.88	
	Alternating gaze	0.02	0.18	0.01
	Group	0.69	0.64	0.14

In neither of the regression models was the interaction between alternating gaze and group significant (*β* = 0.07, *p* > 0.25 with total ADOS-T as dependent variable; *β* = 0.13, *p* > 0.25 with ADOS-T SA as dependent variable; *β* = − 0.29, *p* > 0.25 with ADOS-T RRB as dependent variable); * = *p* < 0.005

**Fig. 4:**
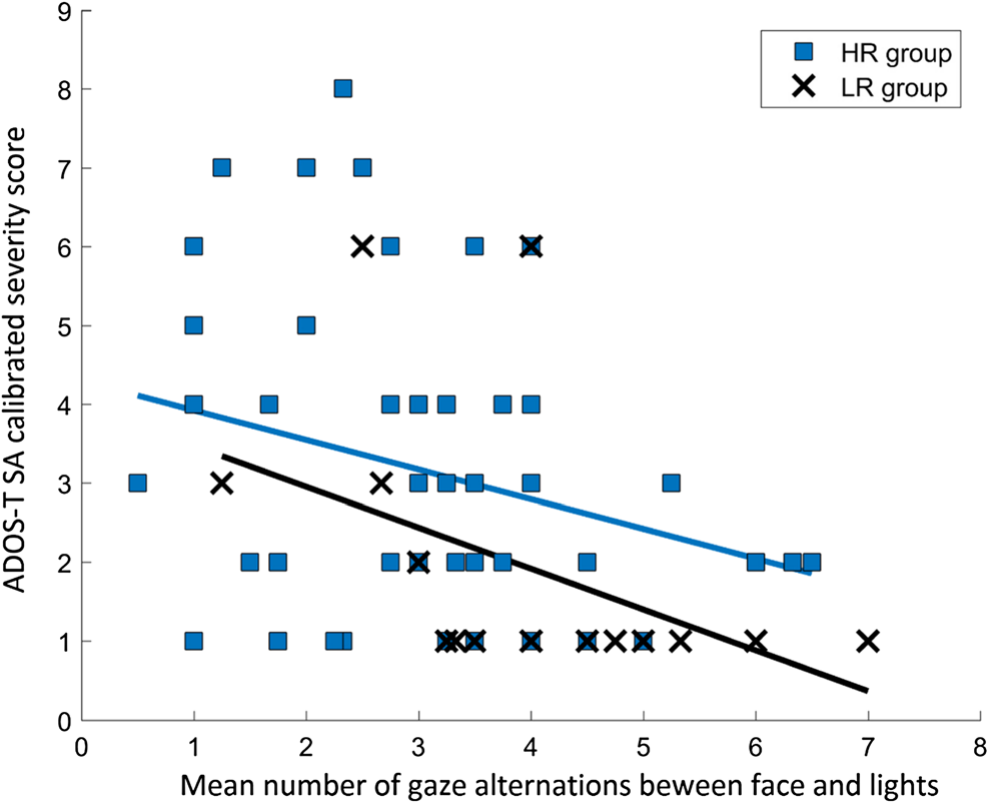
Alternating gaze plotted against ADOS-T SA calibrated severity scores. Figure shows individual data points and separate regression lines for each group

In order to test how alternating gaze was related to the social and the RRB domains, the regression approach described above was repeated twice, using the SA as well as RRB scores as dependent variables. The results of the regression analysis with ADOS-T SA as dependent variable mimicked those of the original regression model, *R*^*2*^ = 0.18, *F*(2,64) = 6.92, *p* = 0.002, whereas the model with ADOS-T RRB as dependent variable was non-significant, *R*^*2*^ = 0.02, *F*(2,64) = 0.61, *p* > 0.25 (Table 3 and Fig. 4). The CSS scores for RRB deviated much more from normality than the other ADOS-T variables (this is a consequence of how the CSS are converted from raw scores in combination with the limited range of scores for this subscale). Therefore, we also conducted non-parametric correlational analyses of the relation between alternating gaze and ADOS-T RRB (Spearman). No associations between alternating gaze and ADOS-T RRB were found in either the HR group, *r*_*s*_(49) = − 0.11, *p* > 0.25, or the LR group, *r*_*s*_(14) = 0.20, *p* > 0.25. In contrast, and in line with the results from the parametric analysis above, significant non-parametric associations between alternating gaze and ADOS-T SA scores were found in both the HR group, *r*_*s*_(49) = − 0.29, *p* = 0.036, and the LR group, *r*_*s*_(14) = − 0.63, *p* = 0.008. Taken together, these analyses indicate that the frequency of gaze alternations at 10 months is related to the social affect domain of ASD, but not to the RRB domain. Consequently, ADOS-T SA rather than total ADOS-T scores will be used as dependent measure in the analyses in the following section.

### Potential Influences of Disengagement and Social Preference on the Relation Between 10-Month Alternating Gaze and 18-Month ADOS-T Social Affect Scores

Although there were no group differences in terms of disengagement or social preference at 10 months, it is still possible that performance on those tasks influences the relation between alternating gaze and later symptom level. To address this issue, another regression analysis was thus performed, including only those infants who had contributed data to both the disengagement task and the social preference task (44 HR infants and 11 LR infants). Again, a bootstrapped hierarchical multiple regression with ADOS-T SA score as dependent variable was conducted, this time entering alternating gaze, disengagement, social preference and group in the first block, and the three interaction terms between those variables and group in the second block. The initial model was significant, *R*^*2*^ = 0.17, *F*(4,50) = 2.62, *p* = 0.046. None of the interaction terms contributed significantly to the ADOS-T SA scores and they were therefore removed from the model. As alternating gaze remained the only variable that uniquely predicted later ADOS-T SA scores, we can conclude that neither disengagement latencies, social preference, nor their interactions with group status influenced the relation between early alternating gaze and later symptomatology. See Table 4.

**Table 4:** Regression table with alternating gaze, disengagement and social preference as independent variables and ADOS-T SA score as dependent variable

	B	SE B	β
Constant	4.31	1.66	
Alternating gaze	−0.51	0.19	−0.41*
Disengagement latency	−1.92	1.20	−0.23
Social preference	2.54	2.42	0.15
Group	0.50	0.67	0.11

None of the interaction terms were significant (alternating gaze*group: *β* = − 0.26, *p* > 0.25; disengagement latency*group: *β* = − 1.28, *p* = 0.215; social preference*group: *β* = 0.96, *p* > 0.25); * *p* = 0.01

### Longitudinal Relations Between 10-Month Alternating Gaze and 18-Month Gestural IJA

In order to investigate whether early alternating gaze was related to later gestural IJA, two bootstrapped hierarchical regression analyses with the ADOS-T items showing and pointing as dependent variables were conducted. As before, alternating gaze and group were entered as independent variables in the first block, and the interaction term was entered in the second block. Both models were significant in the first step (for showing: *R*^*2*^ = 0.17, *F*(2,64) = 6.53, *p* = 0.003; for pointing: *R*^*2*^ = 0.13, *F*(2,64) = 4.83, *p* = 0.011) and as none of the interaction terms were significant, they were removed from the models (Table 5). In both analyses, alternating gaze was the only significant predictor. Following up the regression models with zero-order correlations revealed significant negative correlations between alternating gaze and the ADOS-T item showing in both the HR group, *r*_*s*_(49) = −0.33, *p* = 0.018, and the LR group, *r*_*s*_(14) = −0.50, *p* = 0.047. In other words, infants who engaged more in alternating gaze at 10 months also engaged more in showing at 18 months. For pointing however, only a trend toward a negative correlation with alternating gaze was found in the HR-group, *r*_*s*_(49) = −0.25, *p* = 0.082, indicating that in this group, making fewer gaze alternations at 10 months was associated with less pointing at 18 months. In the LR group, the relation was similar in magnitude and direction, but did not reach statistical significance, *r*_*s*_(14) = − 0.32, *p* = 0.223.

**Table 5:** Regression table with ADOS-T items showing and pointing as dependent variables

Dependent variable		B	SE B	β
Showing	Constant	1.27	0.35	
	Alternating gaze	−0.21	0.07	−0.36*
	Group	0.25	0.25	0.12
Pointing	Constant	1.00	0.30	
	Alternating gaze	−0.16	0.06	−0.33*
	Group	0.15	0.22	0.09

In neither of the regression models was the interaction between alternating gaze and group significant (*β* = 0.28, *p* > 0.25 with showing as dependent variable; *β* = − 0.20, *p* > 0.25 with pointing as dependent variable); * = *p* < 0.05

### Supplementary Analyses

Finally, in order to test the robustness of the results, all analyses were repeated excluding individuals who could be suspected to have influenced the results due to one of two sets of special circumstances, namely explicit initiation of JA during the IJA task (pointing toward or commenting on the lights) or atypically high ADOS-T scores (LR infants only).

In total 5 HR and 1 LR infants made one or more explicit attempts at directing the experimenter’s attention to the lights by pointing toward them or commenting on them. As noted, in these cases, the experimenter responded by turning toward the lights and verbally acknowledging them. Since this opens up for the possibility that the alternating gaze behavior of these infants was affected by the change in experimenter behavior, all analyses were repeated excluding the 6 infants who pointed or vocalized. All results remained the same as before, except for that the group factor now contributed significant variance to the total ADOS-T scores, *β* = 0.23, *p* = 0.011, as well as to the ADOS-T SA scores, *β* = 0.24, *p* = 0.019 (as in the previous analysis, significance levels using bootstrapping are reported).

All data was checked for outliers, and investigating Cook’s distances confirmed that there were no bivariate outliers in any of the analyses. Considering the ADOS scores however, 2 children in the LR group had (total and SA) scores about 2 SD (but <3 SD) above their group’s M. The ADOS is developed for clinical use, and some variation is expected when used in a typical sample. Considering that the 2 LR infants had MSEL scores in the typical range, we do not consider these children outliers. Nevertheless, in order to control for their possible influence on the results, all analyses were repeated removing these 2 children. All significant results remained, but just like in the analysis reported above, group was now a significant predictor of total ADOS-T scores, *β* = 0.29, *p* = 0.001, as well as ADOS-T SA scores, *β* = 0.31, *p* = 0.002.

Excluding infants either because they pointed/vocalized during the IJA task, or because they were LR infants with atypically high ADOS-T scores, thus enhanced the predictive power of the group factor, but did not change the results otherwise.

## Discussion

This study assessed infants’ alternating gaze behavior between an adult and an event occurring outside the adult’s field of vision as a potential measure of early IJA. As hypothesized, we found that 10-month-old infants at high familial risk for ASD made fewer gaze alternations than low risk controls. Moreover, a lower frequency of gaze alternations at 10 months was associated with more core ASD symptoms at 18 months. Thus, alternating gaze behavior can discriminate LR controls from infants at familial risk for ASD, as well as be used to predict later autism symptoms dimensionally. When investigating the relations between alternating gaze and the SA and RRB domains of the ADOS-T separately, they remained significant in both groups in the former case, whereas no relation was detected with the second measure. This suggests that alternating gaze is related to the social symptoms of ASD, but not to restricted and repetitive behaviors.

The majority of the previous studies of early IJA have used more naturalistic methods or clinical instruments. Although these approaches are associated with several advantages - such as high ecological validity - they also have some limitations, one being that it is difficult to control for factors other than IJA that may explain the differences one observes. Taking advantage of precise eye tracking technology, one of the aims of the current study was to investigate the influence of two such potential variables, namely attention disengagement and general social interest. Problems with disengagement of attention have previously been found in children with or at risk for ASD and could result in fewer gaze alternations. Latencies to disengage from one stimulus to another were therefore measured. The performance of the groups did not differ on this task, and disengagement difficulties can thus be ruled out as a cause behind the fewer gaze alternations of the HR group. Longer disengagement latencies were however concurrently associated with less alternating gaze in both groups at 10 months. It is not surprising that infants who take longer to disengage their attention display fewer gaze alternations, as making frequent gaze shifts is dependent on fast disengagement latencies. Importantly though, disengagement latencies were unrelated to later ASD symptoms in the current study. This may be surprising considering that previous studies have reported longer disengagement latencies in infants with a later ASD diagnosis (Elison et al. 2013; Elsabbagh et al. 2013; Zwaigenbaum et al. 2005; Kleberg et al. 2016). This discrepancy may be explained by differences in age between the studies, as none of the other studies measured disengagement at 10 months, but the relation between visual disengagement and ASD clearly warrants further investigation. In terms of social preference, we did not detect either a group difference or any relations with alternating gaze or ASD symptoms. A higher proportion of time spent looking at the experimenter (social preference) and more gaze shifts between experimenter and lights could both reflect a higher social interest or motivation. From that perspective, it may be surprising that our social preference measure was unrelated to both alternating gaze and later ASD symptoms. The reason for this may be that alternating gaze (and IJA), reflects a more active attempt toward sharing experiences with another than does passively looking at social stimuli. IJA by means of gaze alternation has previously been associated with increased activity in the ventral striatum, an area that has been linked to motivation and reward seeking, in a sample of TD adults (Schilbach et al. 2010). It is thus possible that infants who later display more ASD symptoms find attention sharing less rewarding than infants with lower degrees of autistic traits, whereas the same is not necessarily the case for more passive looking patterns.

The study also aimed to clarify the extent to which early alternating gaze is specifically related to established IJA behaviors later on. Looking back and forth between a person and an object is indeed a less explicit manifestation of joint attention compared to the later occurring gestural behaviors, such as pointing and showing. As noted in the introduction, a previous study demonstrated that eye contact based IJA, such as alternating gaze, was concurrently unrelated to gestural IJA in a sample of autistic children (Pickard and Ingersoll 2015). In the current study, a higher frequency of alternating gaze at 10 months was significantly associated with more showing and marginally associated with more pointing at 18 months, suggesting that alternating gaze is longitudinally related to gestural IJA behaviors. As suggested in the introduction, the lack of a correlation in the study by Pickard and Ingersoll (2015) may be related to the age of their participants. The children in that study were 2–7 years old, and language may to some extent have replaced gestural and eye contact based IJA, thus weakening the relation between the two. In the present study we measured both alternating gaze and gestural IJA at ages where language is not yet an effective means of initiating JA for most children. The fact that a longitudinal relation between alternating gaze and gestural IJA was found in the current study strengthens the validity of alternating gaze as an early IJA measure. The measure further has a clear advantage compared to gestural measures in that it allows for earlier use. Pointing, for example, develops quite late with respect to when investigations of IJA are of interest. Although pointing was not the focus of the current study, all instances when infants pointed toward the lights were coded. In total, only three infants pointed during the experiment, rendering inferential statistical testing non-applicable. This confirms that at 10 months, pointing is not a useful measure of individual differences in IJA, as most infants did not display the behavior at all. With the alternating gaze measure, on the other hand, we found substantial individual differences at the same age.

A group difference in alternating gaze was detected already at 10 months, which is very shortly after the first signs of this behavior typically emerges (Beuker et al. 2013). This suggests that the patterns of IJA behaviors in children with later ASD symptoms may be different from the time these behaviors start to develop. Although the current results do not allow us to determine whether reduced alternating gaze is a primary or secondary phenomenon, less engaging in alternating gaze may well impact later development negatively. When the infant initiates joint attention with an adult, the adult is likely to respond by providing information or social interaction. Alternating gaze therefore initiates communication and interaction as well as provides opportunities for learning. Less engagement in IJA early in life could therefore be expected to have extensive consequences on several areas of development. It has recently been suggested that patterns of social looking, which appear to have a substantial genetic component in young children (Constantino et al. 2017), may contribute to so-called evocative gene-environment correlations whereby the child actively shapes its own social environment (Kennedy et al. 2017). Measuring alternating gaze is fast (the current results are based on an experiment that only lasted 1–1.5 min in total) and does not necessarily require eye tracking equipment to be conducted. If the findings are replicated and the task further improved, the alternating gaze measure could thus find practical use in the future. Apart from highlighting the role of alternating gaze in the early development of infants at risk for ASD, this study also contributes information about IJA in typical development. Most studies on IJA and social functioning have focused on atypical populations, which entails that our knowledge about their relation in typical development is rather scarce. In a rare study of typical development, Van Hecke et al. (2007) showed that a higher incidence of IJA at 12 months was related to more optimal social functioning at 30 months. Their and our findings thus both indicate that the relation between alternating gaze and later social functioning is not restricted to clinical samples. This is also compatible with the view that the autistic phenotype represents the extreme end of a continuum, rather than a discrete entity (Lundstrom et al. 2012).

Although the current study is one of the first to use eye tracking to measure IJA, the technology has previously been used for the same purpose by Billeci et al. (2016). In their study, 2-year-olds with and without ASD watched a video of a model seated at a table where a toy car suddenly started to move. In contrast to the current results, it was discovered that the ASD group made *more* gaze alternations between the model and the car than the control group. When comparing the two studies some caution must be given considering that Billeci et al. (2016) focused on diagnosed children, whereas the current study assessed younger siblings. Nevertheless, the discrepancy in results is quite striking, and we can think of at least two possible methodological explanations for this. First, the car used by Billeci et al. (2016) was moving from the periphery toward the center of the screen. It is possible that the TD children interpreted this as the car entering the model’s field of vision, thus rendering alternating gaze in the function of drawing the model’s attention to the car superfluous. In the current study, the flashing lights were outside the model’s field of vision at all times. Second, Billeci et al. (2016) used pre-recorded stimuli whereas the current study was conducted live. The 2-year-old participants in the study by Billeci et al. (2016) could be expected to have an appreciation for the difference between a real person and one appearing on a screen. It therefore remains a possibility that the TD participants’ low incidence of alternating gaze was a consequence of their awareness that the person on screen was not affected by their behavior. The current study highlights the potential as well as the importance of using a more naturalistic approach when studying social behaviors (Falck-Ytter 2015; Falck-Ytter et al. 2015). By applying eye tracking technology in a live setting we were able to combine the advantages of more highly controlled paradigms with those of more ecologically valid ones.

The current study has several limitations that deserve attention. ADOS-T scores were used as an indication of the degree of ASD symptomatology. However, although the ADOS is considered gold standard in ASD assessments, it is not a stand alone instrument that in itself gives a perfect indication of a child’s symptom level. This means, for example, that a high ADOS-T score does not necessarily mean that diagnostic criteria are fulfilled. Thus the current approach should not be confused with one where diagnostic outcome groups are compared, which would require a formal diagnostic assessment. The fact that the two groups differed substantially in size is also a limitation, and even more so is the fact that the LR group consisted of only 16 children (with fewer contributing data to some of the analyses). The conclusions regarding relations within the LR group therefore need to be interpreted with some caution, as well as the conclusion that the relation between alternating gaze and our 18-month measures are similar in both groups. The t-tests that were conducted on the primary and supplementary measures however take the differences in sample sizes into account. Also, it is relatively common to include more HR than LR infants in longitudinal studies (Zwaigenbaum et al. 2005), partially because the HR sample will be split into multiple groups after diagnostic assessment. Importantly, the relation between alternating gaze at 10 months and the total ADOS-T score (explaining 14% of the variance) is based on a reasonable sample size due to pooling of the groups.

### Conclusions

Alternating gaze is one of the first means a young infant has to express and share his/her interest in various objects and events with another person. Looking back and forth between a parent and an object may result in opportunities for social interaction (the parent acknowledges the infant’s behavior by attending to him/her), word learning (the parent labels the object), as well as motoric exploration (the parent hands the infant the object). Alternating gaze is thus a powerful behavior that affects later development in multiple ways. The results of this study indicate not only that infants at risk for ASD engage less in alternating gaze than low risk infants, but also that the tendency to engage in alternating gaze at 10 months is negatively related to social ASD symptoms at 18 months, and that this relation is independent of differences in general social interest and disengagement abilities. The results suggest that a lower tendency to engage in alternating gaze may be one of the earliest signs of ASD in the social domain. Because these early atypicalities may negatively impact patterns of social interaction with caregivers, their precise role in shaping the development of children with ASD should be addressed in future studies.
